# Polymorphism of Drug Resistance Genes *dhfr* and *dhps* in *Plasmodium falciparum* Isolates among Chinese Migrant Workers Who Returned from Ghana in 2013

**DOI:** 10.3390/tropicalmed8110504

**Published:** 2023-11-19

**Authors:** Hong Quan, Peng Yu, Kokouvi Kassegne, Hai-Mo Shen, Shen-Bo Chen, Jun-Hu Chen

**Affiliations:** 1 National Key Laboratory of Intelligent Tracking and Forecasting for Infectious Diseases, National Institute of Parasitic Diseases, Chinese Center for Diseases Control and Prevention (Chinese Center for Tropical Diseases Research), Shanghai 200025, China; 2 National Health Commission of the People’s Republic of China (NHC) Key Laboratory of Parasite and Vector Biology, Shanghai 200025, China; 3 World Health Organization (WHO) Collaborating Center for Tropical Diseases, Shanghai 200025, China; 4 National Center for International Research on Tropical Diseases, Shanghai 200025, China; 5 Dalian Center for Disease Control and Prevention, Dalian 116000, China; 6 School of Global Health, Chinese Center for Tropical Diseases Research, Shanghai Jiao Tong University School of Medicine, Shanghai 200025, China; 7 Hainan Tropical Diseases Research Center (Hainan Sub-Center, Chinese Center for Tropical Diseases Research), Haikou 571199, China

**Keywords:** imported cases, *Plasmodium falciparum*, SP resistance, *dhfr*, *dhps*

## Abstract

In 2013, an epidemic of falciparum malaria involving over 820 persons unexpectedly broke out in Shanglin County, Guangxi Zhuang Autonomous Region, China, after a large number of migrant workers returned from Ghana, where they worked as gold miners. Herein, we selected 146 isolates randomly collected from these patients to investigate the resistance characteristics of the parasite to sulfadoxine–pyrimethamine (SP) by screening mutations in the *dhfr* and *dhps* genes. All 146 isolates were successfully genotyped for *dhps*, and only 137 samples were successfully genotyped for *dhfr*. In the *dhfr* gene, point mutations occurred at three codons: 51 (83.2%, 114/137), 59 (94.9%, 130/137), and 108 (96.4%, 132/137). In the *dhps* gene, mutations occurred at four codons: 436 (36.3%, 53/146 for S436A, 0.7%, 1/146 for S436Y), 437 (95.2%, 139/146), 540 (3.4%, 5/146), and 613 (2.7%, 4/146). All 146 isolates had mutations in at least one codon, either within *dhfr* or *dhps*. Quadruple mutation I_51_R_59_N_108_/G_437_ (41.1%, 60/146) of partial or low resistance level was the most prevalent haplotype combination. Quintuple I_51_R_59_N_108_/G_437_E_540_ accounted for 2.1% (3/146). Sextuple I_51_R_59_N_108_/A_436_G_437_S_613_ was also found and accounted for 1.4% (2/146). A chronological assay incorporating two sets of resistance data from the studies of Duah and Amenga-Etego provided an overview of the resistance trend from 2003 to 2018. During this period, the results we obtained generally coincided with the total development tendency of SP resistance. It can be concluded that *Plasmodium falciparum* samples collected from Chinese migrant workers from Ghana presented prevalent but relatively partial or low resistance to SP. A chronological assay incorporating two sets of data around 2013 indicates that our results possibly reflect the SP resistance level of Ghana in 2013 and that the possibility of increased resistance exists. Therefore, reasonable drug use and management should be strengthened while also maintaining a continuous screening of resistance to SP. These findings also underscore the need to strengthen the prevention of malaria importation from overseas and focus on preventing its reintroduction and transmission in China.

## 1. Introduction

Malaria, a parasitic disease with a significant impact on human health, affects a substantial part of the global population. Consequently, it is regarded as an important public health problem in the modern world. According to the World Health Organization (WHO)’s World Malaria Report 2022, an estimated 247 million cases and 619,000 malaria-related deaths occurred worldwide in 2021 [1]. The WHO African Region accounted for over 90% of cases and deaths.

The Chinese government initiated the National Malaria Elimination Program in 2010 [2], and since then, great progress has been made in malaria control. The incidence of locally acquired malaria cases has continuously declined; however, the number of imported cases has markedly increased [3,4,5]. From 2011 to 2016, the total reported cases of imported malaria reached 20,000, with an average of 3000 each year. The first year during which no indigenous cases appeared in China was in 2017 [6]. All 2861 accumulative reported malaria cases were imported from overseas. From 2017 to 2020, there were four consecutive years during which no indigenous malaria cases were reported within the Chinese territory [7,8,9]. On 30 June 2021, the WHO certified malaria elimination in China, which is a significant milestone for both the public health of China and global progress in malaria elimination [10]. Since then, efforts against malaria in China have shifted towards a defensive strategy aimed at preventing its reintroduction from external sources.

Particularly, in recent years, the growth of international communication and overseas investment has markedly increased personnel mobility to China. This heightened mobility has led to a substantial number of individuals engaging in traveling, business trips, academic exchange, and import and export of labor services [11,12]. These social changes put China under the burden of a large number of imported malaria cases [12,13]. Furthermore, most imported cases were caused by *Plasmodium falciparum* species [13], which can result in severe consequences if not promptly treated. Therefore, imported malaria cases became the main obstacle to the elimination program by 2020 [14].

The main sources of imported malaria cases in China were African and Southeast Asian countries [15]. From late April 2013, a mass of local residents who worked as gold miners in Ghana, a western African country, began to return to their hometown, Shanglin County, Guangxi Zhuang Autonomous Region, China, after the Ghana government adjusted their policy on the gold mining industry. Most of them returned between June and July, and their return led to a large-scale outbreak of malaria in the locality [12]. Considering Ghana’s being one of the main sources of imported malaria cases to China for many years, a large number of-returnees from Ghana in a short time triggered in Shanglin County a rapid and active malaria screen lasting from May to late August by PCR and microscopy on three relevant populations of 6096 persons in total: (1) those with overseas travel histories within 1 year; (2) those with the onset of fever and seeking medical help but without overseas history; and (3) those living with malaria patients. A total of 874 persons were found to be infected with malaria, all of whom had returned from overseas; among them, 871 were from Ghana, and no local malaria cases were found. Of 871 persons from Ghana, 807 were gold miners, and over 820 were infected with falciparum malaria. Artemisinin-based combination therapy (ACT) plus low-dose primaquine against *P. falciparum*, which was responsible for the outbreak, was immediately administered to stop onward transmission. Venous blood was collected from all malaria patients to prepare whole blood samples and filter paper-dried blood drops for storage, based on which a series of laboratory research studies were developed. A relevant drug resistance study on chloroquine and artemisinin revealed that 9.3% and 14.4% of the tested samples were mutated at the chloroquine resistance-related genes *pfmdr1* and *pfcrt*, respectively. Moreover, 8.5% of the samples had mutations in the artemisinin resistance-related gene *K13* propeller [16]. However, a relevant assay has not been conducted for the antifolate drug sulfadoxine-pyrimethamine (SP).

Although abandoned as the first-line anti-malarial drug, SP is still adopted in malaria intermittent preventive treatment during pregnancy (IPTp), mainly in African countries, due to its availability, safety, and effectiveness. Malaria infection in pregnant women in African countries has been a severe and disturbing problem. In 2021, there were approximately 40 million pregnant women in 38 countries of moderate to high malaria transmission in the WHO African region, among whom 13.3 million (32%) were exposed to the risk of malaria infection during pregnancy. The prevalence of malaria infection during pregnancy was the highest (40.7%) in western Africa, slightly lower (39.8%) in central Africa, and 20% in eastern and southern Africa [1]. Malaria during pregnancy will have severe adverse effects on mothers and their offspring, such as maternal anemia, stillbirth, premature delivery, and low birth weight, for which adoption of SP in IPTp can effectively prevent or alleviate them. In addition to IPTp, SP is also used in antenatal care and intermittent preventive treatment in infants (IPTi) and seasonal malaria chemoprevention (SMC) in children under 5 years. Occasionally, it is still used to treat uncomplicated malaria [17,18,19,20,21].

SP resistance has a strong impact on SP effects on IPTp, IPTi, and SMC and severely influences the achievement of malaria prevention in African countries. Therefore, routine monitoring of SP resistance and reasonable use of SP to prevent increasing drug resistance are still very important and must be continued.

For a thorough understanding of the resistance characteristics of this parasite to antimalarial drugs, we selected 146 dried blood spot samples collected from migrant workers returned from Ghana and infected with *falciparum* malaria to investigate the resistance information of the parasite to SP by screening mutations in the *dhfr* and *dhps* genes. The work would help to profile the SP resistance of the parasite, trace the origin and evolutionary pattern, provide beneficial suggestions on future treatment policy, and block entrance and transmission of the highly resistant malaria parasite in China.

## 2. Materials and Methods

### 2.1. Ethics Considerations

This study was conducted in accordance with the principles of the Declaration of Helsinki. Before blood collection, the study protocol and potential risks and benefits were explained to the participants, and written informed consent was obtained from them. Blood samples were collected following the review and approval of institutional ethical guidelines by the Ethics Committee of the National Institute of Parasitic Diseases, Chinese Center for Disease Control and Prevention.

### 2.2. Sample Collection and DNA Extraction

From over 820 dried blood spot samples on FTA filter paper collected in 2013 from migrant workers from Ghana to Shanglin County, Guangxi Zhuang Autonomous Region, China, who were infected with falciparum malaria, we selected 146 isolates by random in our study to investigate the SP resistance of *P. falciparum* by screening the *dhfr* and *dhps* genes.

Genomic DNA samples were extracted from dried blood spots on FTA filter paper using a QIAamp DNA Blood Mini Kit (250) (Qiagen, Hilden, Germany) according to the manufacturer’s instructions.

### 2.3. PCR Amplification and Sequencing

The amplification of *dhfr* and *dhps* gene fragments was performed via nested PCR as previously described [19,22]. The primer sequences used in the PCR amplification are listed in Table 1. For both *dhfr* and *dhps* fragments, the primary amplification was performed as follows: initial denaturation at 95 °C for 5 min, followed by 35 cycles of denaturation at 95 °C for 30 min, annealing at 50 °C for 30 min, extension at 68 °C for 1 min, and a final extension at 68 °C for 5 min. The product of the primary procedure was subjected to the nested one with the same amplification conditions except for the annealing at 52 °C and the cycling number of 30. The amplified PCR products were purified using Biomagnetic beads/Silicic modified (Henan Huier Nano Technology Co., Ltd., Zhengzhou, China) and sequenced using the Sanger method (Genewiz INC, South Plainfield, NJ, USA) with both primers. The sequencing results were aligned against the *pfdhfr* (Accession Number; XM_001351443) and *pfdhps* (Accession Number; XM_001349382) 3D7 reference sequences published in the NCBI database using Mega 7.0.21 to assay specific mutation sites. All amplification and sequencing processes were conducted twice, and repeated verification was carried out if different results were obtained in two runs.

### 2.4. Chronological Analysis of SP Resistance

A chronological assay was performed using two sets of resistance data from approximately 2013 to observe the results over several consecutive years.

The samples in our study were collected from Chinese migrant workers who returned from Ghana in 2013. Because these patients were infected naturally in Ghana territory, the SP resistance characteristics of the sample could be regarded as a reflection of the national situation in Ghana in 2013, disregarding the racial factor. It is important to note that we only had access to these samples for 1 year. Using the year 2013 as the center point, we adopted the data from the studies of Duah [21] and Amenga-Etego [23] spanning from 2002 to 2018. We organized data from these three sources chronologically to observe the development of SP resistance over a span of 16 years and determined if our findings align with the overall trend.

We consolidated the findings of Duah’s study conducted in nine sentinel sites in Ghana and adopted the results as the SP resistance data in Ghana from 2003 to 2010 (Table 2). Furthermore, the results of Amenga-Etego’s study conducted in northern Ghana served as the data source for the period spanning 2009 to 2018.

## 3. Results

### 3.1. Mutations in the dhfr Gene

**Figure 1:**
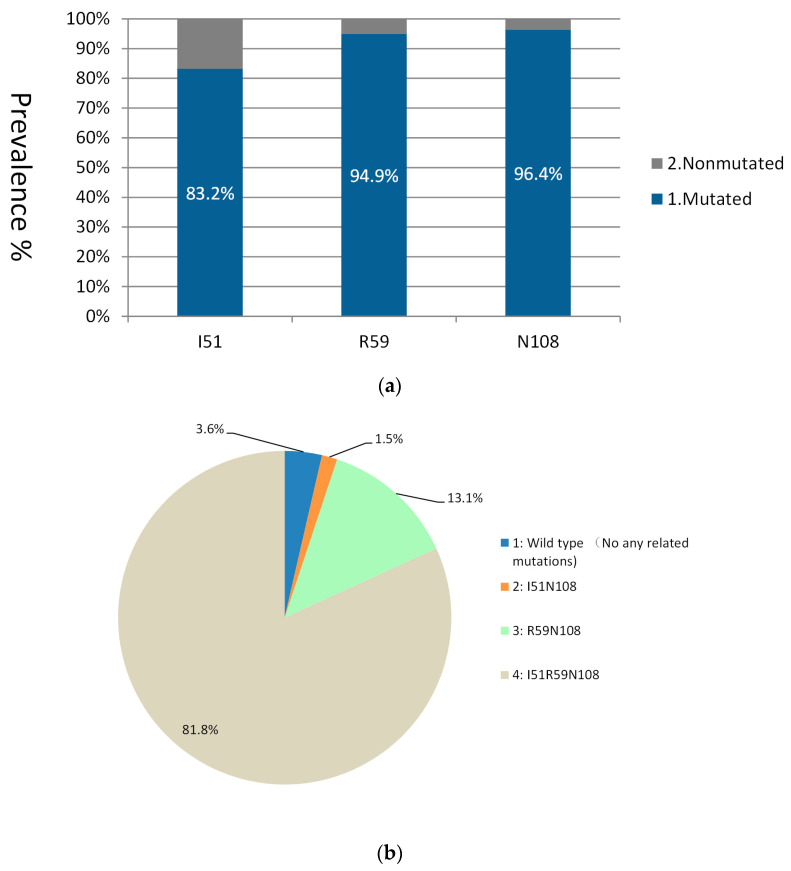
Prevalence of single point mutations and genetic haplotypes in the *dhfr* gene of the 137 isolates analyzed. (**a**) Prevalence of single point mutations in the gene locus of the *dhfr* gene. (**b**) Genetic haplotype in the *dhfr* gene.

Among 146 samples, 137 (93.8%, 137/146) were successfully genotyped for the *dhfr* gene covering codon positions 16, 51, 59, 108, and 164. Point mutations were detected at three codons—51, 59, and 108—with a prevalence of 83.2% (114/137), 94.9% (130/137), and 96.4% (132/137), respectively (Figure 1a). No mutant alleles were detected at codons 16 and 164. Four *dhfr* haplotypes were observed. The triple mutation I_51_R_59_N_108_ was the most prevalent (81.8%, 112/137) (Figure 1b).

### 3.2. Mutations in the dhps Gene

**Figure 2:**
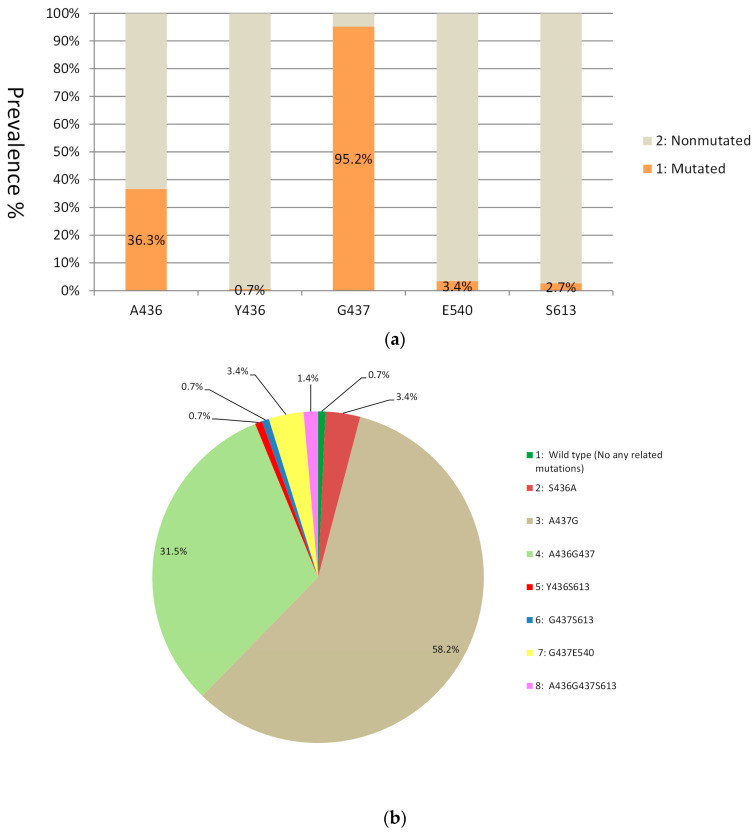
Prevalence of single point mutations and genetic haplotypes in the *dhps* gene of the 146 isolates analyzed. (**a**) Prevalence of single point mutations in the gene locus of the *dhps* gene. (**b**) Genetic haplotype in the *dhps* gene.

All 146 samples were successfully genotyped for the *dhps* gene (100%, 146/146). Point mutations occurred in four sites and produced five alleles: S436A (36.3%, 53/146), S436Y (0.7%, 1/146), A437G (95.2%, 139/146), K540E (3.4%, 5/146), and A613S (2.7%, 4/146) (Figure 2a). Eight *dhps* haplotypes were observed. G_437_ was the most prevalent (58.2%, 85/146). G_437_E_540_ accounted for 3.4% (5/146) (Figure 2b).

### 3.3. dhfr/dhps Haplotype Combination

All 146 samples had mutations in at least one site, either in the *dhfr* or *dhps* gene. In total, 15 *dhfr*/*dhps* haplotype combinations were observed. I_51_R_59_N_108_/G_437_ was the most prevalent, with 41.1% (60/146), whereas I_51_R_59_N_108_/G_437_E_540_ accounted for 2.1% (3/146) (Table 3).

### 3.4. Chronological Analysis of SP Resistance

The chronological arrangement of SP resistance data from our study and the studies of Duah and Amenga-Etego is shown in Table 2.

Through data organization, we gained a primary understanding of the development tendency of SP resistance in Ghana over a 16-year period, spanning from 2003 to 2018. Notably, mutations of *dhfr* I_51_, R_59_, N_108_, and *dhps* G_437_ increased steadily during this period. In contrast, the mutation at codon 540 of *dhps* remained unchanged or was at a very low level. Furthermore, *dhps* S613 remained at 3.4–19% from 2009 to 2018, as reported in Amenga-Etego’s study.

For haplotypes, *dhfr* IRN increased from 2009 and stabilized at approximately 80%. In contrast, the *dhps* G_437_E_540_ level was generally very low each year.

For haplotype combination, quadruple of I_51_R_59_N_108_/G_437_ or I_51_R_59_N_108_/A_436_ decreased from 2009 to 2018; however, quintuple of I_51_R_59_N_108_/A_436_G_437_ or I_51_R_59_N_108_/G_437_E_540_ increased significantly from 2015 and reached the highest value of 88.6% in 2016, as reported in Amenga-Etego’s study.

## 4. Discussion

More than 20 years ago, chloroquine, the first-line anti-malaria drug of the first generation since the 1970s, faced widespread resistance to *P. falciparum* in many parts of the world. SP was introduced as a replacement for chloroquine as the first-line treatment against chloroquine-resistant *falciparum* malaria [24,25,26,27]. SP is a synergistic antifolate combination used alone or in combination with other anti-malaria drugs. SP-based therapy was initially effective, but resistance gradually emerged. The first case of SP resistance was on the Thailand–Cambodia border in the 1960s and was reported by Bjorkman and Phillips-Howard [28]. Since then, almost all countries have modulated their malaria prevention policy by adopting ACT as a first-line anti-malarial therapy as recommended by the WHO, including African countries in the sub-Saharan region [25]. The emergence of SP resistance occurred earlier than its replacement as the first-line drug, and it lasted only 4 years before SP was replaced by ACT [24].

Although there is currently no first-line antimalarial drug, SP remains the only option for IPTp, especially in African countries. Aside from IPTp, SP is also used as an IPTi and SMC and sometimes as a partner drug of ACT, impacting its efficacy with regard to SP resistance. Therefore, continuous screening of SP resistance is still indispensable [17,29]. Furthermore, being no longer the first-line treatment for more than 10 years would raise the possibility of decreased SP resistance in theory [25]. However, unlike the reemergence of chloroquine susceptibility after the removal of drug pressure [30], SP resistance seems to be stabilized in Africa. The stabilization may be attributable to the use of SP for IPTp [25]. Until now, the *falciparum* parasite has developed resistance to nearly all anti-malaria drugs, including artemisinin and its derivatives, which are very effective against malaria parasites. The reason for continuous screening of SP resistance is not solely related to IPTp usage of SP but also pertains to the possibility of restoring SP sensitivity. If SP sensitivity is restored after a long period of nonuse, it can once again play an active role in malaria control.

SP resistance is monitored by screening mutations in the *dhfr* and *dhps* genes of *P. falciparum*. N_108_ and G_437_ are the cores of the *dhfr* and *dhps* mutations, respectively, both indicating only a very low grade of resistance. The intensity of the resistance increases progressively with the addition of mutations at the other codons: 16, 51, 59, and 164 for N_108_ and 436, 540, 581, and 613 for G_437_. The I164L mutation, which often occurs together with the *dhfr* triple mutation I_51_R_59_N_108_, stands for pyrimethamine resistance of hardly the highest grade and leads to the rapid spread of antifolate resistance [17,31,32]. Mutations at codons 540, 581, and 613 cannot occur alone; they usually appear in association with those at 437 and/or 436. Mutations at codons 540 and 581 indicate full or super resistance [33].

The extent of SP resistance is determined by varying numbers and combinations of mutations present in the *dhfr* and *dhps* genes. The *dhfr* triple mutation I_51_R_59_N_108_, indicating a very low grade of SP resistance, is common in Africa. The *dhfr* I_51_R_59_N_108_ plus *dhps* G_437_ was associated with SP resistance in West Africa. Quintuple mutation I_51_R_59_N_108_/G_540_E_613_ is more common in East and Southern Africa than West Africa, and it stands for full or intense resistance to SP, which can increase the risk of SP treatment failure up to 75% [17,31,32]. Furthermore, the acquisition of G_581_ and/or L_164_ confers the parasite with super resistance. SP-IPT in infants and pregnant women is reported to have failed in super-resistant areas [33]. The WHO recommends that where the prevalence of E_540_ exceeds 50%, SP-IPT in infants should not be implemented [33].

In our study, all 146 isolates exhibited mutations in at least one site, either within the *dhfr* or *dhps* gene. Five isolates (3.7%) retained the wild-type status of the *dhfr* gene, and only one isolate (0.7%) retained the wild-type status of the *dhps* gene. No mutation was observed at codons 164 and 581. I_51_R_59_N_108_/G_437_ of partial or low resistance level was the most prevalent at 41.1%. Of the isolates, 2.1% presented quintuple mutations of I_51_R_59_N_108_/G_437_E_540_. The sextuple haplotype involving codon 613 was observed in 1.4% of the samples. All these results indicated a high prevalence of SP resistance in our samples, but at partial or low resistance levels.

In 2005, Ghana changed its malaria control policy by replacing chloroquine with ACTs and adopting SP for IPTp [21]. For other parts of the world, SP is still being used to treat uncomplicated malaria in non-pregnant patients, except for IPTp, and this has led to increased SP resistance. Duah et al. reported a significant increase in the prevalence of mutations in the *P. falciparum dhfr* and *dhps* genes from 2003 to 2010, spanning 2 years before and 5 years after policy change [21].

Subjecting our data and those from the studies of Duah and Amenga-Etego to a chronological analysis of SP resistance provides an overview of SP resistance trends in Ghana during a consecutive time period, spanning from 2003 to 2018. This also demonstrated the alignment of our data with the broader context of malaria in Ghana, highlighting similarities in SP resistance patterns within the context of native infections.

The results of our analysis offer an understanding of the development of SP resistance in Ghana over a period of 16 years. Mutations in *dhfr* I_51_, R_59_, N_108_, and *dhps* G_437_ increased steadily during this period. Colon 540 of the *dhps* gene retained the wild-type status or was mutated at a very low level. *Dhps* S613 remained at 3.4–19% from 2009 to 2018, as reported in Amenga-Etego’s study. For haplotypes, *dhfr* I_51_R_59_N_108_ increased from 2009 and stabilized at approximately 80%. In contrast, the *dhps* G_437_E_540_ level was generally very low for each year. For haplotype combination, quadruple of I_51_R_59_N_108_/G_437_ or I_51_R_59_N_108_/A_436_ decreased from 2009 to 2018; however, the quintuple of I_51_R_59_N_108_/A_436_G_437_ or I_51_R_59_N_108_/G_437_E_540_ significantly increased from 2015 to the highest value of 88.6% in 2016, as reported in Amenga-Etego’s study. However, the apparent increase in the quintuple mutant may be attributed to more instances of I_51_R_59_N_108_/A_436_G_437_ and fewer occurrences of I_51_R_59_N_108_/G_437_E_540_ due to the low or nonexistent occurrence of the E_540_ mutation during this period.

Therefore, the chronological assay indicates that our results align with those reported from 2003 to 2018 on the SP resistance trend. SP resistance was prevalent but at a relatively partial or low level. However, the existence of stabilized S_613_ values for many years and the appearance of S_613_-associated sextuples indicate the possibility of a worsening situation.

The alignment of our results with those of Duah and Amenga-Etego was somewhat unreasonable due to the sample differences. Compared to over 1000 samples in the studies of both Duah and Amenga-Etego, we only had 146 isolates in our study. The time spans of sample collection were 7 and 9 years for Duah and Amenga-Etego, respectively, while our samples were collected only within a very short time of a single year of 2013. The number of imported malaria cases has markedly increased in China since 2010, but only in 2013 did we have a chance to collect a large number of falciparum malaria samples in a sudden epidemic outbreak. For the infected personnel, we had no detailed information about their ages, but most of them were gold miners and young adults who had returned from Ghana. Furthermore, 146 isolates were enough in number to interpret mutations in the *dhfr* and *dhps* genes and the SP resistance characteristics of malaria parasites of Ghana origin. We collected the same samples of more quantity, on which the relevant study will be carried forward.

Long-term use of a drug against the malaria parasite is likely to lead to the development of resistance. Chloroquine and SP, the first-line anti-malaria drugs of two generations, have been successfully replaced with ACT. However, ACT resistance conferred by mutations in the *P. falciparum k13* gene has gradually emerged, and this has introduced a new challenge to malaria control [34].

To summarize, reasonable use of SP in IPTp and other fields is very important for maintaining drug efficacy, and continuous screening of SP resistance is still necessary in Ghana. The prevention of imported malaria cases from overseas is of great significance for China following the successful elimination of malaria.

## 5. Conclusions

*P. falciparum* samples collected from Chinese migrant workers who returned from Ghana presented prevalent resistance to SP but at relevant partial or low resistance levels. A chronological assay incorporating two sets of data from approximately 2013 indicates that our results possibly reflect the level of SP resistance in Ghana during that year. Moreover, there is a possibility of increased resistance in the future. Therefore, reasonable drug use and management should be strengthened while also maintaining a continuous screening of resistance to SP. These findings underscore the need to strengthen the prevention of malaria importation from overseas and focus on the prevention of reintroduction and transmission of the disease in China.

## Figures and Tables

**Table 1:** Primer sequences for amplification of the *dhfr* and *dhps* gene fragment.

*dhfr*	First round	5′-TCCTTTTTATGATGGAACAAG-3′
		5′-AGTATATACATCGCTAACAGA-3′
	Second round	5′-TTTATGATGGAACAAGTCTGC-3′
		5′-ACTCATTTTCATTTATTTCTGG-3′
*dhps*	First round	5′-AACCTAAACGTGCTGTTCAA-3′
		5′-AATTGTGTGATTTGTCCACAA-3′
	Second round	5′-ATGATAAATGAAGGTGCTAG-3′
		5′-TCATTTTGTTGTTCATCATGT-3′

**Table 2:** Chronological arrangement of SP resistance data from our study and the studies of Duah and Amenga-Etego.

Gene			Year													
			Duah’s Study				Our Study	Amenga-Etego’s Study								
			2003– 2004	2005– 2006	2007– 2008	2010	2013	2009	2010	2011	2013	2014	2015	2016	2017	2018
*dhfr*	Point allele	I_51_	49	49.1	60.8	60.7	83.2	-	-	-	-	-	-	-	-	-
		R_59_	52.2	61.9	71.9	80.9	94.9	-	-	-	-	-	-	-	-	-
		N_108_	69.7	68.9	76.6	80.9	96.4	-	-	-	-	-	-	-	-	-
	Haplotype	IN	9	11.1	7.6	3.4	1.5	3.3	2.2	1.8	6.1	1.2	1.1	5.7	4.8	2.2
		RN	15	22	17.3	21.2	13.1	7.5	9.2	14.5	6.1	20.7	18.5	7.1	16.1	24.6
		IRN	35.3	28.8	46.1	53.9	81.8	80	77.2	76.4	66.7	67.5	73.6	79.8	70.7	67.9
*dhps*	Point allele	G_437_	59.2	71.5	80	77.6	95.2	2.5	33.7	9.1	39.4	55.4	64	71.2	69.5	78.2
		E_540_	0	0.5	0.2	1.1	3.4	0	0	0	3.0	0	1.7	1.1	1.3	0
		S_613_	-	-	-	-	2.7	14.2	19	18.2	6.1	7.1	3.4	3.4	4.1	6.9
	Haplotype	G_437_E_540_	0	0.5	0.2	1.1	3.4	0	0	0	3.0	0	1.7	1.1	1.3	0
		A_436_G_437_S_613_	-	-	-	-	1.4	2.5	3.8	3.6	6.1	2.5	9.6	4.6	8.0	4.2
*dhfr/dhps* haplotype combination	Triple ^1^		-	-	-	-	7.5	3.5	0	0	0	5.5	1.4	1.3	0	2.8
	Quadruple ^2^		-	-	-	-	50.7	43.9	49.4	87.5	59.1	45.2	25.7	10.1	21.1	20.6
	Quintuple ^3^		-	-	-	-	30.1	52.6	50.6	12.5	40.9	49.3	72.9	88.6	78.9	76.6
	Sextuple ^4^		-	-	-	-	1.4	-	-	-	-	-	-	-	-	-

^1^: R59N108/A436 for Amenga-Etego’s study; R59N108/G437 for our study. ^2^: I51R59N108/G437 or I51R59N108/A436 for Amenga-Etego study; I51R59N108/G437, I51R59N108/A436, R59N108/A436G437, R59N108/G437E540, R59N108/G437S613, I51N108/A436G437 and I51N108/G437E540 for our study. ^3^: I51R59N108/A436G437 or I51R59N108/G437E540 for Amenga-Etego study; I51R59N108/A436G437, I51R59N108/G437E540 and I51R59N108/Y436S613 for our study. ^4^: I51R59N108/G437E540G581 for Amenga-Etego’s study, but no data available; I51R59N108/A436G437S613 for our study.

**Table 3:** Prevalence of *dhfr*/*dhp* haplotype combinations in the Ghana sample.

*dhfr*/*dhps* Haplotype Combinations		Prevalence % (n)
Triple	R_59_N_108_/G_437_	7.5 (11/146)
Quadruple	I_51_R_59_N_108_/G_437_	41.1 (60/146)
	I_51_R_59_N_108_/A_436_	3.4 (5/146)
	R_59_N_108_/A_436_G_437_	3.4 (5/146)
	R_59_N_108_/G_437_E_540_	0.7 (1/146)
	R_59_N_108_/G_437_S_613_	0.7 (1/146)
	I_51_N_108_/A_436_G_437_	0.7 (1/146)
	I_51_N_108_/G_437_E_540_	0.7 (1/146)
Quintuple	I_51_R_59_N_108_/A_436_G_437_	27.4 (40/146)
	I_51_R_59_N_108_/G_437_E_540_	2.1 (3/146)
	I_51_R_59_N_108_/Y_436_S_613_	0.7 (1/146)
Sextuple	I_51_R_59_N_108_/A_436_G_437_S_613_	1.4 (2/146)

## Data Availability

All materials and data supporting these findings are contained within the manuscript. The sequences have been deposited in the GenBank database under the accession numbers OR264676-OR264812 for the *dhfr* gene sequences and OR269469-OR269614 for the dhps gene sequences from the field isolates collected from the Ghana-imported cases in China.
